# Cross‐Cultural Beliefs and Stigmatization in Vitiligo: A Systematic Review

**DOI:** 10.1111/jocd.70725

**Published:** 2026-03-24

**Authors:** Sophia Ma, Tarek Zieneldien, Isabella J. Tan, Mohammad Jafferany

**Affiliations:** ^1^ School of Medicine Johns Hopkins University Baltimore Maryland USA; ^2^ Robert Wood Johnson Medical School, Rutgers The State University of New Jersey Piscataway New Jersey USA; ^3^ Department of Psychiatry and Behavioral Sciences Central Michigan University College of Medicine Mount Pleasant Michigan USA

**Keywords:** cross‐cultural beliefs, cultural perceptions, psychodermatology, psychosocial distress, quality of life, stigmatization, vitiligo

## Abstract

**Background:**

Vitiligo is an autoimmune condition marked by depigmentation of the skin and is frequently associated with psychosocial distress. Although often dismissed as cosmetic, vitiligo carries a substantial burden influenced by cultural beliefs, stigma, and access to medical education.

**Aims:**

This review aims to examine the literature on cross‐cultural beliefs, stigmatization, psychological comorbidities, and quality of life (QoL) outcomes in individuals with vitiligo.

**Methods:**

A comprehensive search of PubMed, Embase, and PsycINFO was conducted to identify peer‐reviewed studies discussing cultural beliefs, stigma, psychological burden, or QoL in individuals with vitiligo. Inclusion criteria encompassed original studies in English assessing relevant psychosocial or cultural domains.

**Results:**

Twenty‐three studies met inclusion criteria. Cultural attributions of vitiligo varied widely, with some populations linking the disease to contagion, divine punishment, or supernatural causes. These beliefs were more prevalent in regions with lower health literacy and limited access to dermatologic care. Stigmatization—manifesting as social exclusion, employment and marital discrimination, and internalized shame—was consistently reported across settings, though more severe in female patients and individuals with darker skin phototypes in certain regions. QoL impairment was greater in African, Middle Eastern, and South Asian populations compared to Western cohorts. Coping strategies included concealment, spiritual reliance, and use of complementary and alternative medicine (CAM).

**Conclusion:**

Vitiligo imposes a global psychosocial burden that is amplified by cultural misconceptions and stigma. Culturally tailored, multidisciplinary interventions—including education, psychological support, and community‐based stigma reduction—are crucial to improving outcomes. Future research should focus on high‐stigma, underrepresented populations to inform equitable care.

**Abbreviations**
BPRS: Brief Psychiatric Rating Scale
CAM: complementary and alternative medicine
DLQI: Dermatology Life Quality Index
QoL: quality‐of‐life
RSES: Rosenberg Self‐ Esteem Scale

## 1 Introduction

Vitiligo, a chronic inflammatory autoimmune condition characterized by the progressive loss of melanocytes, results in well‐demarcated areas of skin depigmentation. It commonly affects visible bodily areas such as the hands and face, significantly impacting psychosocial well‐being and quality of life [1, 2]. Vitiligo is linked to polymorphisms in genes that regulate melanogenesis and immune function, but environmental triggers are required for clinical expression [2]. Lesions may develop at any age, but the onset typically occurs at or before 30 years of age [3]. The global prevalence is estimated at 0.5%–2.0%, with geographic variation observed [4].

Vitiligo is often misinterpreted as a cosmetic condition, yet patients experience a greater burden than healthy individuals, as shown by quality‐of‐life (QoL) measures [5, 6]. The QoL burden associated with vitiligo may be significantly influenced by coexisting psychosocial morbidities, such as depression, anxiety, and low self‐esteem, as well as related challenges with sexual functioning and finding employment [6, 7]. However, the degree of psychosocial impact and stigma experienced by individuals with vitiligo is shaped by cultural beliefs, societal attitudes, and religious ideologies, which vary across different geographical regions and communities [8]. Understanding these cross‐cultural influences is essential for developing culturally sensitive interventions, assessing the global burden of vitiligo, and promoting person‐centered care. The objective of this systematic literature review was to examine cross‐cultural beliefs and stigmatization in vitiligo, with a focus on stigma, mental health, and QoL outcomes.

## 2 Methods

A comprehensive literature search of the PubMed, Embase, and PsycInfo databases was conducted from inception to June 22, 2025 using the following search terms: (vitiligo) AND (stigma OR stigmatization OR discrimination OR beliefs OR attitudes OR perceptions OR misconceptions OR myths OR psychosocial OR psychological OR “mental health” OR “quality of life” OR QoL) AND (culture OR cross‐cultural OR “cultural beliefs” OR “cultural differences” OR “cultural attitudes” OR “cultural perceptions” OR ethnicity OR “ethnic groups” OR race). Studies were included if they discussed individuals living with vitiligo and explored aspects related to stigma, psychological burden, cultural beliefs, mental health, or quality of life, they were written in English and published in peer‐reviewed journals, and were a relevant study type (i.e., clinical trials, case–control studies, cohort studies, case reports, case series, cross‐sectional studies). Given the relative scarcity of large‐scale randomized psychosocial studies specifically addressing cultural beliefs and stigma in vitiligo, case series and case reports were included when they provided original, contextually relevant qualitative data, contributing to a more holistic understanding of the sociocultural and psychological dimensions of the condition. Exclusion criteria consisted of studies deemed irrelevant to the topic, incorrect publication types (i.e., letters to the editor, review articles, viewpoints, commentaries, study protocols, position statements, conference abstracts), and studies that were published in a language other than English. Articles were independently screened by two reviewers. All disagreements were resolved through discussion between the reviewers to ensure accurate and appropriate inclusion.

## 3 Results

### 3.1 Search Results

**FIGURE 1:**
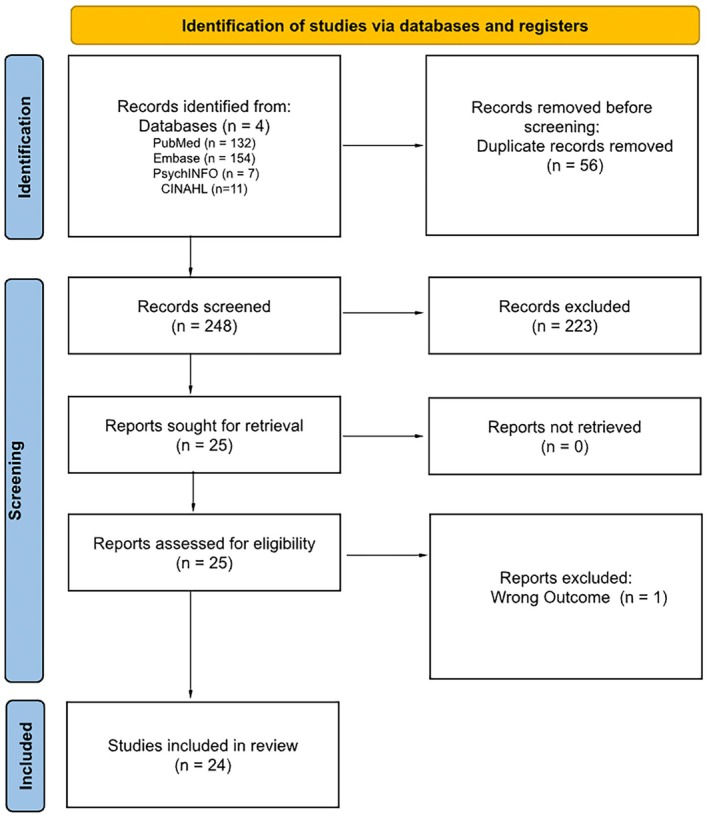
PRISMA flow diagram for new systematic reviews illustrating the number of records identified, screened, and included for each step.

A comprehensive literature search yielded a total of 304 records: 132 from PubMed, 154 from Embase, 7 from PsycINFO, and 11 from CINAHL (Figure 1). Following the removal of 56 duplicate entries, 248 records remained for screening on the basis of the predefined inclusion and exclusion criteria. Of these, 70 were excluded because of inappropriate article type, 148 were removed for having irrelevant titles or abstracts, 2 were excluded for being in a non‐English language, and 3 were deemed unrelated to the research topic. One article was excluded because of reporting an outcome that was not relevant to the review. Ultimately, 24 full‐text articles were retrieved, evaluated for eligibility, and included in the review.

### 3.2 Quantitative Findings

#### 3.2.1 Cultural Beliefs, Myths and Misconceptions

Vitiligo is a condition contextualized within sociocultural frameworks that are influenced by varying beliefs often shaped by local traditions, religious values, and collective experiences [9] (Table 1). In some South Asian, African, and Middle Eastern societies, vitiligo is often perceived not only as a medical ailment, but may also be attributed to curses, divine punishment, or spiritual impurity. Likewise, a cross‐sectional study reported that Arab vitiligo patients attributed their condition to biological factors like stress and heredity, as well as supernatural causes such as fate or the evil eye, whereas 8% believed the disease was contagious [10]. Despite some overlap in beliefs, knowledge of and attitudes toward vitiligo vary among different ethnic groups. In contrast, another study found that 90% of Turkish vitiligo patients believed their condition was not contagious, and 80% reported no negative impact on their relationships with family or friends [11]. In a multi‐ethnic population study conducted in Thailand, participants' perceptions were assessed after viewing a video demonstration about vitiligo. The results showed that Arabians achieved the highest knowledge scores regarding vitiligo, whereas Caucasians had the highest proportion of participants with a positive attitude, followed by Arabians, Asians, and Africans [12]. Among Asian and African participants, those with sufficient knowledge about vitiligo were more likely to have positive attitudes toward individuals affected by the condition [12].

**TABLE 1:** Summary of included articles discussing vitiligo beliefs, stigma, and psychological consequences across regions.

Study (Author, Year)	Population demographic/region	Study focus	Key findings	Study type (*n* = number of vitiligo patients)
AlGhamdi 2010	Arab vitiligo patients	Beliefs about vitiligo	Widespread misconceptions and negative attitudes; depression and anxiety comorbidities	Cross‐sectional observational study (*n* = 164)
Jean‐Pierre et al. 2024	African American and Hispanic/Latino vitiligo patients in the United States	Mental health burden	African American and Hispanic/Latino vitiligo patients had a higher mental health burden than Caucasian vitiligo patients	Retrospective analysis and cross‐sectional study (*n* = 1087; 212 African American, 257 Hispanic/Latino, 531 Caucasian)
Juntongjin et al. 2022	Asian, Caucasian, Arabian, and African participants in Bangkok, Thailand	Vitiligo awareness, knowledge, and attitudes in the general population	Knowledge of and attitudes toward vitiligo varied across ethnicities, 30% of the participants could identify vitiligo	Cross‐sectional survey (*n* = 405 non‐patient participants; 193 Asian, 95 Caucasian, 94 Arabian, 23 African)
Mattoo et al. 2002	Indian vitiligo patients	Psychiatric morbidity	25% of vitiligo patients had psychiatric morbidity, most commonly adjustment disorder	Case–control, observational study (*n* = 113)
Papadopoulos et al. 2002	Pakistani, Caucasian, Afro‐Caribbean, Indian vitiligo patients in the United Kingdom	Vitiligo illness beliefs	Internal and external causal attributions vary across race and gender	Cross‐sectional survey (*n* = 922)
Pahwa et al. 2013	Indian vitiligo patients	Psychosocial impact of vitiligo	Vitiligo causes major physical, social, and emotional challenges, especially for women	Semi‐structured interviews (*n* = 50)
Pichaimuthu et al. 2011	Indian vitiligo and psoriasis patients	Stigma and social participation	17.3% of vitiligo patients had limited participation in domestic and social activities.	Cross‐sectional comparative study (*n* = 300 total patients; 150 vitiligo, 150 psoriasis patients)
Poondru et al. 2024	Vitiligo patients in the United States	Complementary and alternative medicine usage by vitiligo patients	32.5% of participants reported previous CAM usage; non‐white participants are more likely to use CAM	Cross‐sectional survey (*n* = 625; 425 Caucasian, 53 Asian, 47 African American, 59 Hispanic, 28 Mixed, 6 Other)
Porter et al. 1991	African American and Caucasian vitiligo patients in the United States	Stigma and disturbance degree	African Americans experience more stigma from racial identity, Caucasians from appearance; stigma raises distress in both	Cross‐sectional observational study (*n* = 158; 93 Caucasian, 65 African American)
Sangma et al. 2015	Indian vitiligo patients	Psychosocial impact and morbidity	Vitiligo significantly worsens the quality of life and heightens depression compared with healthy individuals	Cross‐sectional, case–control study (*n* = 100 vitiligo patients, *n* = 50 matched controls)
Spratt 2022	Vitiligo and alkaptonuria patients in the United States	Coping	Vitiligo patients use passing, performative, and resistance strategies to manage stigma and social interaction, shaped by racial divisions.	Semi‐structured interviews (*n* = 34 total)
Strouphauer et al. 2024	African American pediatric vitiligo patients in the United States	Prevalence of psychiatric comorbidities and treatment initiation	More likely to be diagnosed with depression, disruptive behavior disorders, eating disorders, generalized anxiety disorder, substance abuse, and suicidal ideation.	Retrospective, single‐center, case–control study (*n* = 327 vitiligo patients, 981 controls)
Taylor et al. 2024	Nigerian vitiligo patients	Experience of living with vitiligo	Patients experienced stigma but found support through faith and community	Participatory interpretative phenomenological analysis/semi‐structured interviews (*n* = 8)
Thompson et al. 2010	British South Asian female vitiligo patients	Stigma and living with vitiligo	Vitiligo leads to stigma and identity challenges due to cultural views on appearance	Semistructured interviews (*n* = 7)
Thompson et al. 2022	Caucasian, Black, British South Asian heritage vitiligo patients in the United Kingdom	Mental health associations	Higher incidence of recurrent depressive and anxiety disorder in vitiligo patients, especially in Black and minority ethnic populations	Retrospective observational cohort study (*n* = 7224 vitiligo patients, 28 880 controls; 3504 Caucasian, 1060 Asian/Asian British, 170 Black/African/Caribbean/Black British, 238 Mixed, 1115 Other vitiligo patients)
Topal et al. 2016	Turkish vitiligo patients	Knowledge, beliefs, and perceptions on vitiligo	74% of patients had knowledge of their condition, and did not view vitiligo as having a major negative impact	Cross‐sectional, descriptive, prospective study (*n* = 100)

Additionally, patients often seek out complementary and alternative medicine (CAM), influenced by cultural beliefs that promote spiritual or natural remedies, as well as diet or lifestyle myths. In the United States, 32.5% of individuals with vitiligo reported using CAM, with common choices including vitamin supplements and *Nigella sativa* oil [13]. Non‐White participants were more likely than White participants to use CAM and held more favorable perceptions toward these therapies [13].

#### 3.2.2 Stigmatization and Social Consequences

Vitiligo patients across cultures commonly face distressing experiences of stigma, social isolation, and discrimination. In a study of Arab patients, 42% believed their condition significantly affected others' perceptions of them [10]. A cross‐sectional study in India found that 17.3% of vitiligo patients experienced significant social and domestic participation restrictions, reflecting considerable stigma in this population [14]. In the United States, African American vitiligo patients reported experiencing greater stigma than their White counterparts and expressed heightened concerns about how depigmentation might affect their racial identity [15]. These consistent patterns across diverse settings highlight the pervasive social consequences of vitiligo.

#### 3.2.3 Psychological Comorbidities and Daily Living Impairment

Individuals with vitiligo experience a substantial burden of psychological comorbidities and daily living impairment across diverse cultural settings. Case control studies indicate that psychiatric morbidity affects approximately 25% of vitiligo patients in India, most commonly as adjustment disorders, and may also present with higher depression rates and decreased QOL [16, 17]. In contrast, in the United Kingdom, patients show an increased risk of depression and anxiety but not adjustment disorders, with the highest vulnerability observed among Black and minority ethnic populations–a pattern also identified in the United States [18, 19]. Similar trends are evident in Arab, where depression and anxiety are particularly prevalent among female patients, and among African American pediatric populations in the United States, who demonstrate elevated rates of depression, disruptive behavior, and eating disorders compared to controls [10, 20].

Furthermore, studies across different demographic regions indicate that vitiligo markedly impairs QoL, though the extent varies across cultural and regional contexts. Patients in Africa, the Middle East, Malaysia, and Central India commonly report moderate to severe QoL impairments, whereas those in Western and European populations tend to experience milder effects, as reflected by Dermatology Life Quality Index (DLQI) scores (Table 2). Moreover, the impact of factors such as gender roles and skin phototype on the severity of QoL impairment differs across regions, with women and patients with darker skin tones experiencing greater distress in certain areas (Table 2).

**TABLE 2:** Summary of quality of life assessments among vitiligo patients across various cultural regions.

Study (Author, Year)	Vitiligo patient population demographic/region	Number of participants (*n*)	Assessment tool	QoL impairment score
Al‐Mubarak et al. 2011	Saudi	26	Questionnaire: 41 questions grouped into 4 dimensions (relation with colleagues, family life, social relationships, self‐respect)	Mean = 17.1; 11.1 for males, 23.9 for females (*p* < 0.05)
Bibeau et al. 2022	European, Japanese, American	219 (Europe, *n* = 150; United States, *n* = 48; Japan, *n* = 21)	VitiQoL	Median = 48 (IQR = 18–70). No difference for ethnicity, worse scores for greater extent of disease and patients 25–44 years
Ingordo et al. 2014	Italian	161	DLQI	Mean = 4.3 ± 4.9; DLQI > 5 associated with female gender, disease stability, and facial involvement at onset
Kent et al. 1996	United Kingdom	614 (non‐Caucasian *n* = 46, Caucasian = 567)	DLQI, version of Ginsburg and Link's psoriasis stigma questionnaire, Rosenberg's scale, symptom checklist, and 12‐item General Health Questionnaire	DLQI mean = 4.82 ± 4.84; non‐Caucasian (7.78) reported higher levels of disability than Caucasians (4.59); no difference between males and females; DLQI scores related to perceived stigma, recent experiences, self‐esteem, personal distress, and race
Khan et al. 2023	Central Indian	115	DLQI, Rosenberg Self‐ Esteem Scale (RSES), and Brief Psychiatric Rating Scale (BPRS)	DLQI: mean = 12.38 ± 4.5 RSES: 16.4 ± 2.9 BPRS: 28.47 ± 4.8; 18–30 years of age group had the highest DLQI score, lowest RSES, and highest BRPS
Kiprono et al. 2013	Tanzanian, Southern Kenya	88	DLQI	Mea*n* = 7.2 ± 4.8
Raheel et al. 2024	Female North African	65	Skindex‐16 scale	Mea*n* = 2.998 ± 0.505; QoL differed with age, social and functional status, and economic status
Sangma et al. 2015	North‐East India	100	DLQI, Hamilton depression rating scale (HAMD‐17)	DLQI: mean = 9.08 ± 4.46 (controls: 1.04 ± 1.12) HAMD‐17: 8.45 ± 3.44 (controls: 3.20 ± 2.43)
Wong et al. 2012	Malaysian (Malay, Chinese, Indian, others)	102 (Malay, *n* = 45; Chinese, *n* = 20; Indian, *n* = 35; Others, *n* = 2)	DLQI	Mean = 6.40 ± 5.17; Malays 7.51 ± 5.18, Chinese 6.6 ± 5.99, Indians 5.00 ± 4.55; Malays had higher DLQI scores compared to Indians (*p* = 0.026)

### 3.3 Qualitative Findings: Cross‐Cultural Beliefs and Stigmatization

Several qualitative studies provide rich contextual insights into the experiences and cultural perceptions of individuals with vitiligo. In a participatory interpretative phenomenological analysis, Nigerian vitiligo patients reported both personally experiencing and friends or family fearing that bad spirits or a curse was the cause of the disease [19]. All participants interviewed attributed coping with vitiligo to prayer and God [19]. Similarly, semi‐structured interviews among Indian vitiligo patients revealed diverse attributions for vitiligo, including diet deficiency, injury, medication reaction, sickness, environmental changes, infections, burns, bites, stress, anger, internal body issues, or divine will [21]. A proportion of the patients and their friends or family believed the disease was contagious, heritable, or related to leprosy [21].

Patients also engaged with traditional healing practices, such as consulting Ayurvedic doctors or indigenous medicine practitioners, following dietary restrictions of specific foods, and avoiding wearing certain materials [21]. Community support strategies included forming ethic‐ or race‐based support groups that fostered a shared cultural understanding and resilience [22]. Nigerian patients similarly reported seeking support within church communities, attributing their coping to faith in God [23].

Experiences of stigmatization and discrimination were common across settings. Nigerian patients described social exclusion and discrimination [23]. Indian patients reported ostracism, social restriction, employment difficulties, and marriage challenges felt more acutely by women [14, 21]. British South Asian women echoed similar stigmatization experiences, including family exclusion, concealment, cultural disconnection, and concerns about marital prospects [24]. This qualitative evidence highlights culturally specific beliefs and social consequences shaping the psychosocial burden of vitiligo in diverse populations.

### 3.4 Divergent Cultural Beliefs and Stigmatization Patterns Across Regions

Across studies, a clear gradient exists between regions with high medical literacy and those with limited dermatologic education: Western and urban populations generally reflect biomedical understanding of vitiligo, whereas rural or traditional communities often sustain spiritual or moral explanations [10, 12, 14, 19, 21]. In sub‐Saharan Africa, vitiligo may be associated with witchcraft or ancestral displeasure [19, 23], whereas in parts of India and the Middle East, the condition is linked to dietary taboos, “hot‐cold” imbalances, or impurity ideology embedded in religious practice [10, 21]. These explanatory models strongly shape help‐seeking behaviors, where patients may first consult spiritual healers, traditional practitioners, or religious figures before seeking dermatologic care [13, 21, 23].

A similar reliance on CAM and traditional practices has been observed across Asia and Africa. Herbal preparations, avoidance of “trigger foods,” and ritual cleansing are frequently viewed as essential components of healing [14, 21, 23]. Such practices reflect the enduring influence of culturally rooted treatment paradigms that coexist with, or sometimes substitute for, evidence‐based dermatologic care.

Corresponding to these explanatory beliefs, the intensity and form of stigma also differ by region. In South Asian societies, where marriageability and aesthetic norms carry strong social weight, vitiligo is often perceived as a barrier to matrimony and family acceptance, particularly for women [21, 23]. In sub‐Saharan Africa, affected individuals report overt exclusion, avoidance in workplaces and markets, and misconceptions equating vitiligo with leprosy or other transmissible diseases [19, 23]. In Western populations, stigma tends to be more subtle and internalized, reflected in social anxiety, self‐consciousness, and concealment rather than overt social exclusion [15, 23].

Gender and skin phototype further modulate these experiences: women and individuals with darker skin tones frequently report greater visibility‐related distress, reduced quality of life, and compounded discrimination [10, 15, 23, 24, 25]. The intersection of gender, cultural beauty norms, and pigmentation visibility thus amplifies psychosocial burden.

Finally, community responses also exhibit notable cultural variation. Some patients in collectivist settings describe supportive kinship or religious networks that frame vitiligo as a test of faith, whereas others experience stigmatizing pity or social withdrawal [23, 24]. These divergent responses highlight the complex interplay between cultural belief systems, disease visibility, and community integration.

## 4 Discussion

The results of the review underscore how individuals with vitiligo are profoundly affected by culturally rooted beliefs and stigma, which differ across regions and contribute to a varied quality of life and psychological comorbidities. Across multiple societies in South Asia, Africa, and the Middle East, vitiligo is often attributed to spiritual or supernatural causes– such as curses, divine punishment, or spiritual impurity– rather than being recognized as a formal medical condition [10, 19, 21]. Furthermore, some people mistakenly believe vitiligo is contagious or that the condition originates from emotional disturbances or disease [21]. These beliefs persist despite medical evidence, particularly in communities where health literacy is low or where patients lack accurate information about the disease or its curability [26, 27, 28]. These culturally‐rooted misconceptions perpetuate stigma and discrimination, shaping both individual and societal perceptions, especially in cultural contexts where visible differences are closely scrutinized or stigmatized [24]. Despite these overarching myths, knowledge and attitudes toward vitiligo vary widely: although most Turkish patients correctly recognize that vitiligo is not infectious, many in South Asia and the Middle East continue to harbor myths about its cause and transmission [10, 11, 21]. Addressing these deeply rooted misconceptions is crucial, not only to reduce the psychological and social burdens experienced by those with vitiligo but also to promote acceptance and evidence‐based care on a global level.

Cross‐cultural differences in vitiligo‐related beliefs and stigma reveal distinct sociocultural determinants that shape patient experiences across regions [10, 11, 12, 13, 14, 15, 19, 21, 22, 23, 24]. In South Asian and Middle Eastern contexts, stigma is often rooted in religious or supernatural attributions, such as curses, divine punishment, impurity, or contagion, reflecting the influence of collectivist social structures and moralized interpretations of visible disease [10, 14, 19, 21]. In sub‐Saharan African settings, beliefs frequently invoke spiritual causation or ancestral curses, and stigma is reinforced by limited access to dermatologic education and persistent disease myths within community and church networks [19, 23]. By contrast, in European and North American populations, vitiligo is predominantly understood as a biomedical or autoimmune disorder, yet psychological morbidity persists, often driven by appearance‐based distress, racial identity concerns, and colorism, particularly among individuals with darker phototypes [15, 18, 24].

These findings suggest that although stigma is universal, its social expression and internalization are culture‐specific, governed by local visibility norms, gender expectations, and explanatory models of disease [10, 19, 21, 23, 24]. Further, intra‐regional variation is evident: urban and medically literate communities typically demonstrate greater disease understanding and reduced stigma, whereas rural or traditional populations retain misconceptions and supernatural attributions [10, 12, 14]. This highlights that “culture” is not monolithic but stratified by socioeconomic, educational, and religious subcontexts, influencing how vitiligo is perceived and managed within the same geographic area.

Finally, geographic representation within the published literature remains uneven, with limited data from sub‐Saharan Africa, Latin America, and Southeast Asia. As such, whereas the observed patterns are consistent across available studies, they cannot fully represent global diversity. Future work should prioritize underrepresented regions and incorporate non‐English‐language and community‐based studies to strengthen generalizability and inform culturally responsive stigma‐reduction interventions [26, 27, 28].

These cultural variations also extend to coping mechanisms and treatment‐seeking behaviors, which are shaped by local belief systems and social expectations [13, 21, 23, 24]. Coping mechanisms among vitiligo patients are also culturally mediated and vary accordingly, but avoidance, concealment, and reliance on spiritual support are common themes [23, 24]. Many patients also turn to CAM, including natural remedies, dietary adjustments, religious interventions, or indigenous medicines, reflecting a blending of traditional values with treatment [13, 21]. These findings highlight the need for culturally sensitive, multidisciplinary interventions to provide satisfactory treatment for both dermatological and psychological aspects of vitiligo.

Stigma remains a widespread and distressing consequence for individuals living with vitiligo. Across cultures, stigmatization, decreased self‐esteem, and negatively impacted QoL are prevalent and are further compounded by gender, cultural attitudes, and vitiligo lesion visibility [10, 15, 23, 24, 25]. These social challenges often translate into depression and anxiety and psychological comorbidities, especially in minority ethnic groups [10, 19, 20, 29].

These findings underscore a need for culturally sensitive educational initiatives to dispel enduring myths about vitiligo and to promote accurate understanding in both healthcare settings and the wider community. Healthcare practitioners should be attentive to the cultural contexts that influence patient beliefs and behaviors and should offer support through interventions that address the distinct social and psychological needs of diverse populations. Although some misconceptions about vitiligo are widespread, variation in beliefs and experiences across regions and cultures highlights the need for context‐specific approaches. Effectively addressing the challenges faced by individuals with vitiligo requires a comprehensive approach that integrates culturally informed psychosocial support and targeted community education in order to improve QoL and reduce stigma.

## 5 Study Limitations

This review is limited by heterogeneity in study design, population demographics, and outcome measures, which restricts cross‐study comparability. Most studies were either cross‐sectional or qualitative, limiting causal inferences between cultural beliefs, stigma, and psychosocial outcomes. Although the inclusion of case reports and series may lower the overall level of evidence, this approach was important to adequately capture the holistic diversity of sociocultural contexts and lived experiences represented in the literature. Assessment tools for quality of life and psychological burden were often non‐validated or lacked cultural adaptation, potentially affecting accuracy and generalizability.

Although gray literature and trial registries were not searched, the review covered the major biomedical and psychosocial databases (PubMed, Embase, PsycINFO, and CINHAL), which were selected for their strong indexing of dermatology, psychiatry, and cross‐cultural research. The decision aimed to maintain a focus on peer‐reviewed, methodologically sound studies while balancing feasibility and thematic relevance. Nevertheless, the exclusion of non‐indexed or unpublished studies may have introduced publication bias favoring positive or English‐language results.

Geographic representation was also uneven, with underrepresentation of populations from sub‐Saharan Africa, Latin America, and Southeast Asia. The exclusion of non‐English publications may have introduced language bias and limited regional perspectives.

## 6 Conclusion

Vitiligo is a chronic autoimmune disease with a significant psychosocial burden, shaped by cultural beliefs, gender norms, skin phototype, and access to health education. This review demonstrates that stigma, social exclusion, and psychological comorbidities–particularly depression, anxiety, and low self‐esteem–are prevalent across diverse populations, though their expression and severity vary by sociocultural context. Misconceptions such as contagion, divine punishment, and spiritual impurity persist in many regions and may contribute to reduced quality of life, difficulties with employment and marriage, emotional distress, and cosmetic dissatisfaction.

Populations with greater awareness–demonstrated in Turkey and parts of Europe–report lower stigma and milder QoL impairment, highlighting the protective role of medical education. Coping strategies often include concealment, avoidance, spiritual reliance, and community support, with increased use of CAM observed in certain cultural groups.

There is a need for multidisciplinary, culturally tailored interventions that integrate dermatologic care with psychosocial support and stigma reduction. Future research should prioritize underrepresented and high‐stigma populations to inform equitable models of care.

## Funding

The authors have nothing to report.

## Ethics Statement

The authors have nothing to report.

## Conflicts of Interest

The authors declare no conflicts of interest.

## Data Availability

Data sharing not applicable to this article as no datasets were generated or analysed during the current study.
